# New Appliances and Things Medical

**Published:** 1902-10-11

**Authors:** 


### NEW APPLIANCES AND THINGS MEDICAL.

**[We shall be glad to receive at our Office, 28 & 29 Southampton Street, Strand, London, W.C., from the manufacturers, specimens of all new preparations and appliances which may be brought out from time to time.]**

### NATURAL CARLSBAD SALTS.

(Sole Importers for Great Britain and the Colonies: Ingram and Royle, Ltd., 26 Upper Thames Street, London, E.C.)

These natural Carlsbad salts, which bear the trade mark and signature of "Karlsbader Mineral wasser versendung Löbel Schottlander," consist of the genuine saline constituents of the Sprudel springs at Carlsbad. They thus differ from certain other salts, which, though passing under the name of Carlsbad, are really combinations of the similar salts, but derived from quite a different source. In the preparation of the above natural salts the following method is adopted. The Sprudel water is superheated, this causes a free liberation of carbonic acid and the precipitation of manganese, lime, iron, and magnesium carbons; these salts which are useless for therapeutical purposes for which the Carlsbad waters are ordered, are removed by filtration, and the filtrate evaporated until it is reduced to a residue of semi-solid consistency, it is then saturated with the carbonic acid so freely given off at the Sprudel springs. The object of this saturation is to re-transform the mono-carbonates of lithia and soda into bicarbonates, thus bringing them back to the original condition as they occur in the spring water. With the exception that the earthy carbonates have been removed, the dried Carlsbad salts when re-dissolved in water present exactly the same chemical combination as occurs in the Sprudel spring. It is unnecessary to enter into the therapeutic properties of these salts; but in all cases in which a "cure" at Carlsbad is indicated, the taking of these salts at home offers a good substitute for the use of the waters. The Natural Carlsbad Salts are prepared in crystalline and in powder form. The dose is one teaspoonful taken either in hot or cold water the first thing in the morning.

### THE "ICEBERG" BUTTER BOX.

(Dairy Supply Company, Limited, Museum Street, London, W.C.)

By a curious misuse of words the term "refrigerator" has been applied to various boxes made of non-conducting materials, by the use of which things placed within them can be kept cool. In this sense the "Iceberg" Butter Box is a refrigerator, that is to say, it will keep its contents cool so far as its thickness keeps out the heat. It is a very simple contrivance, being, indeed, only a well-made box containing one or more trays of tin, with divisions, on which to place the butter. Still, even so simple a contrivance as this, granting that it is well made is of great use in preserving a commodity like butter, which is not only peculiarly susceptible to changes of temperature but is particularly receptive in regard to the odours and volatile matters which are so often met with in ordinary larders. That it will quite do all that is claimed for it is perhaps rather hard to believe, but that it will do a great deal towards keeping butter sweet and in good condition, that it will keep the butter clean and that it will prevent it taking to itself the odours which arise from the various kinds of food which in small houses are stuffed into the same larder, is very certain. Like everything else it will itself require to be kept clean, but if this be done, and if the cook can be persuaded to shut it at once every time she takes a bit of butter out, it will certainly be found a most useful article.

